# Serum Treatment of Plague

**Published:** 1915-10

**Authors:** 


**Serum Treatment of Plague.** J. Guiteras and Alberto Recio, Sanidad and Beneficencia, Havana, November and December, 1914. Haffkine's vaccine and Paris Serum were used in this epidemic. Twenty-seven cases of plague occurred and six died. The results seem to speak favorably for the effect of the Paris serum.

In twelve cases treated in the Las Animas Hospital, all recovered but one. None of them were unusually mild cases. The buboes suppurated in all of them save one. The beneficial influence of the serum appeared to be striking.

Dr. Racio reports as follows:

Treatment. As far as our present knowledge goes, it appears to be demonstrated that the only successful treatment of plague is to be found in specific serum therapy. That however, the real value of antiplague sera appears difficult to determine with precision. In the case that died at the Las Animas Hospital rather small doses were given intravenously. Seven cases were treated at the Covadonga Hospital with two deaths. Of these two, one was admitted in a state of great gravity, about the fourth day of the disease and died three hours after the administration of the serum. The second case did not present characteristic symptoms of the disease on admission and was overlooked until the third day after admission and the fourth day of the disease.

The quality of the serum is of great importance. The serum used in these cases showed itself to be sufficiently active and when applied opportunely, and in sufficient doses, never failed to give evidence of its action.

In all cases they employed large doses at intervals of 6, 12 and 24 hours, according to the necessities of the case.

The method if administration used by Recio is as follows:

Injection into a vein of 80 c.c. **immediately** upon the confirmation of the diagnosis.

Repetition of the same dose six hours later if the fall of temperature be not initiated. If this has occurred he delays the injection 12 hours.

If the descent continues the next dose of 80 c.c. is given twenty-four hours later.

Subsequent doses of 40 c.c. are given every twenty-four hours until the temperature is brought completely under control.

The treatment is discontinued when the temperature occilates about normal. About 200 to 600 c.c. of serum are required.

Duprat, in Rio Grande do Sul, in 1902 used larger doses. His first dose, according to the gravity of the case was from 200 to 300 c.c. subcutaneously. He continued with doses of 100 to 150 c.c. every 12 hours. He obtained 38 cures in 45 cases treated.

Choksy uses Lustig's serum, as follows:
Injection of 60, 80 or 100 c.c. as soon as the diagnosis is made. Children under 12 years of age, 10 c.c. An injection intravenously of 20 to 30 c.c. as recommended by Calmette may be employed.

Injections are given in the morning and repeated in 24 hours. If the patient is seen first in the afternoon, the injection is made at once and repeated in the morning.

The amount of serum injected will depend on the rise of temperature the previous evening and the condition of the patient. For the same temperature, the same amount of serum; for a lower temperature, 30 c.c. less serum.

The quantity of serum should be lessened gradually until the temperature falls to normal in the mornings.

A sudden fall of temperature between the second and the seventh days should not indicate a suspension of the treatment.

The injections are given every 12 hours if secondary buboes present themselves, or if the temperature rises rapidly one or two degrees.

If the evening temperature be lower than the morning, the dose is reduced the following day.

From six to eight injections are generally sufficient to complete the treatment.

The total quantity of serum varies from 150 to 300 c.c. according to the gravity of the symptoms and the condition of the serum.

While it must be inferred from the above facts that the last word has not yet been spoken on the subject, still the results are encouraging, and the able investigations of such men as Guiteras, Lebredo, Recio and others will, in the end, prevail, and rob the Black Plague of many of its horrors.

